# Limited IgM seroreactivity to arboviruses in free-ranging black-tufted marmosets (*Callithrix penicillata*)

**DOI:** 10.3389/fvets.2026.1827761

**Published:** 2026-08-13

**Authors:** Daniel Oliveira dos Santos, Bruna Hermine de Campos, André Duarte Vieira, Letícia Neves Ribeiro, Caio de Castro Cunha Figueiredo, Janaína Ribeiro Duarte, Vinícius Henrique Barbosa Amaral, Lucas dos Reis de Souza, Nayara Ferreira de Paula, Maira Alves Pereira, Sara Cândida Ferreira dos Santos, Felipe Campos de Melo Iani, Marcelo Pires Nogueira de Carvalho, Ayisa Rodrigues de Oliveira, Renato Lima Santos

**Affiliations:** 1 Departamento de Clínica e Cirurgia Veterinárias, Escola de Veterinária, Universidade Federal de Minas Gerais, Belo Horizonte, Brazil; 2 Fundação Ezequiel Dias, Belo Horizonte, Brazil

**Keywords:** chikungunya, dengue, serology, yellow fever, zika

## Abstract

Arboviruses are important causes of human disease, and several species also infect wild animals that may serve as amplifying hosts. Among these, yellow fever virus (*Orthoflavivirus flavi*) is a major cause of morbidity and mortality in neotropical primates. In addition to yellow fever virus, several other arboviruses have been reported in these animals. For example, zika virus (*Orthoflavivirus zikaense*) has been detected in free-ranging marmosets and capuchin monkeys. Antibodies against dengue virus (*Orthoflavivirus denguei*) have been identified in *Alouatta* spp. and *Ateles* spp., while *Sapajus* spp., *Ateles marginatus*, and *Callithrix jacchus* have been shown to develop neutralizing antibodies against chikungunya virus (*Alphavirus chikungunya*). In this study we assessed anti-arboviruses (*A. chikungunya*, *O. zikaense*, *O.Denguei*, and *O. flavi*) antibodies in free-ranging black-tufted marmosets (*Callithrix penicillata*) living in urban parks. Frequency of putative seroreactivity was low with 2.94% (2/68) of the captured marmosets seroreactive to *A. chikungunya* (IgM) and 1.47% (1/68) seroreactive to *O. zikaense* (IgM). One marmoset was seroreactive to both viruses (*A. chikungunya* and *O. zikaense*). Arboviruses genomic sequences were not detected in serum samples, but seroreactive animals were not tested by RT-PCR due to sample limitation. This study demonstrated limited evidence of putative seroreactivity of free-ranging black-tufted marmosets to arboviruses in an area where these viruses are endemic in the human population.

## 1 Introduction

Arboviruses are transmitted by arthropods and cause great impact in human health. Several viruses belonging to the *Orthoflavivirus* genus are included in this group such as yellow fever virus (*Orthoflavivirus flavi*), dengue virus (*Orthoflavivirus denguei*), Saint Louis virus (*Orthoflavivirus louisense*), West Nile virus (*Orthoflavivirus nilense*), and zika virus (*Orthoflavivirus zikaense*). Relevant arboviruses also include chikungunya virus (*Alphavirus chikungunya*), an *Alphavirus* (1, 2). These viruses are transmitted either between humans via an invertebrate vector or from wild or domestic animals to the vector, with these animals serving as amplifying hosts that facilitate subsequent human infections (3).

Yellow fever is an important disease associated with outbreaks in human and non-human primate populations (4). More than 2,000 human cases occurred during the last outbreak in Brazil from 2016 to 2019 (5). Neotropical primates were greatly impacted during this outbreak with reduction of free-ranging populations of threatened species such as golden lion tamarins (*Leontopithecus rosalia*) and northern muriquis (*Brachyteles hypoxanthus*) (6, 7). Severity of disease for neotropical primates varies according to the affected species that may develop severe necrotic hepatitis, whereas other species may develop no histopathological lesions upon infection with *O. flavi* (8, 9).

Other arboviruses have been investigated in neotropical primates: squirrel monkeys (*Saimiri* sp.), owl monkeys (*Aotus* sp.), and common marmosets (*Callithrix jacchus*) develop viremia and antibodies after experimental infection with *O. zikaense* (10, 11), and natural infections with *O. zikaense* has been reported in free-ranging marmosets and capuchins as diagnosed by molecular and serological methods (12, 13). *O.Denguei* seroreactivity was reported in howler monkeys (*Alouatta* sp.) and spider monkeys (*Ateles* sp.) (14, 15). Neutralizing antibodies against *A. chikungunya* have been detected in capuchins (*Sapajus* sp.), white-cheeked spider monkeys (*Ateles marginatus*), and common marmosets (*Callithrix jacchus*) (16).

Black-tufted marmosets (*Callithrix penicillata*) are small neotropical primates highly adapted to urban environments (17). Marmosets can host *O. flavi* and *O. zikaense* and there is serological evidence of natural infection with *A. chikungunya* in this species (8, 12, 16). Since neotropical primates can act as sentinels for yellow fever, mainly howler monkeys (*Alouatta* sp.), and other arboviruses can infect neotropical primates, the goal of this study was to investigate putative seroreactivity of free-ranging black-tufted marmosets (*C. penicillata*) to arboviruses.

## 2 Methods

### 2.1 Ethics statement

All procedures were authorized by the Ethics Committee on the Use of Animals of the Universidade Federal de Minas Gerais (CEUA/UFMG) under protocol number 78/2022, by Instituto Chico Mendes de Conservação de Biodiversiade under protocol number 81392, and by Fundação de Parques Municipais e Zoobotânica de Belo Horizonte (FPMZ-BH) under protocol numbers FU002/2022, and Sisgen under protocols ABB00DB and ACDCB02.

### 2.2 Animal procedures

**Figure 1:**
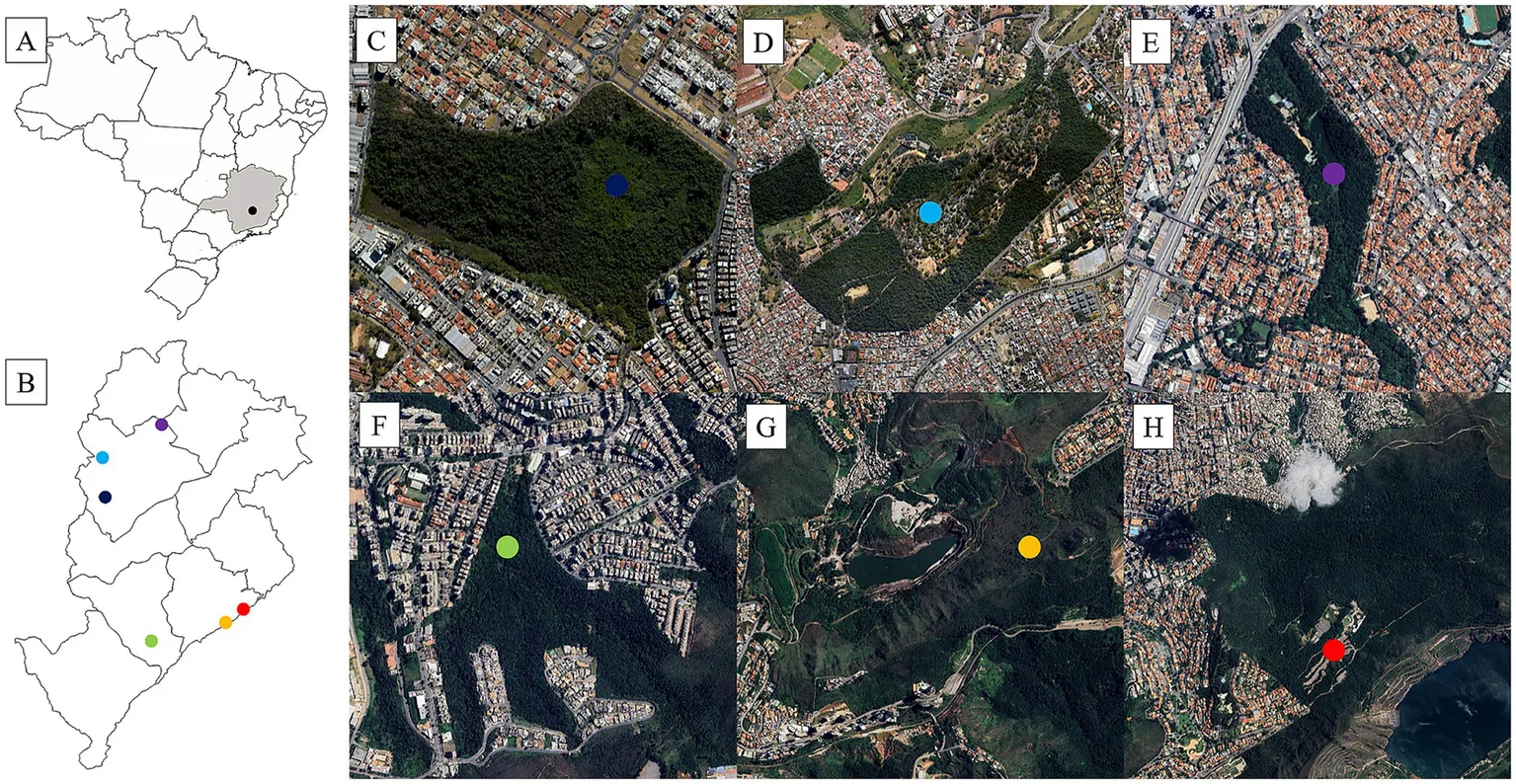
Areas of capture of free-ranging black-tufted marmosets (*Callithrix penicillata*) in Belo Horizonte (Minas Gerais, Brazil). **(A)** Map of Brazil with the State of Minas Gerais in gray and location of Belo Horizonte (black dot). **(B)** Belo Horizonte map with various colored dots (areas of capture). **(C–H)** Satellite view of each area of capture. **(C)** *Parque Ursulina de Andrade Mello* (U; dark blue dot; number of captures/animals [*n*] = 5/5). **(D)** Belo Horizonte Zoo (Z; light blue dot; *n* = 10/10). **(E)** *Parque Fazenda Lagoa do Nado* (LN; purple dot; *n* = 15/14). **(F)** *Parque Aggeo Pio Sobrinho* (AP; green dot; *n* = 2/2). **(G)** *Parque da Serra do Curral* (SC; orange dot; *n* = 4/4). **(H)** *Parque das Mangabeiras* (PM; red dot; *n* = 36/33). Maps Data: Google, © 2014.

Black-tufted marmosets (*C. penicillata*) were captured in six parks in the city of Belo Horizonte, Brazil (Figure 1): *Parque Aggeo Pio Sobrinho* (AP; area: 612,346.68 m^2^; altitude: 850–1,050 m); *Parque Fazenda Lagoa do Nado* (LN; area: 313,101.10 m^2^; altitude: 780–810 m); *Parque das Mangabeiras* (PM; area: 2,547,732.49 m^2^; altitude: 904–1,375 m); *Parque da Serra do Curral* (SC; area: 408,071.95 m^2^; altitude: 945–1,509 m); *Parque Ursulina de Andrade Mello* (U; area: 307,174.65 m^2^; altitude: 830–870 m); and Belo Horizonte Zoo (Z; area: 1,008,414.31 m^2^; altitude: 815–909 m). Captures took place from 2022 and 2023 with two capture periods per year, from January to June and from July to December. The first period of capture (2022.1) was a pilot that took place only at PM. The number of capture time points varied according to the parks: 1 capture at AP (2023); 3 at LN (1 in 2022 and 2 in 2023); 4 at MP (2 in 2022 and 2 in 2023); 2 at SC (1 in 2022 and 1 in 2023); 3 at U (1 in 2022 and 2 in 2023); and 3 at Z (1 in 2022 and 2 in 2023). In total, there were 16 capture time points between 2022 and 2023 in six different parks. In total, 4 animals were recaptured (2 captures for each). The samples of these recaptured animals were tested independently.

Traps were baited with bananas, distributed in multiple points and checked twice a day. Captured animals were weighted and sedated with ketamine (IM, 20 mg/kg) and midazolam (IM, 1 mg/kg). During sedation, animals were clinically evaluated, sex determined and age estimated. Clinical evaluation consisted of external exam, body condition, superficial lymph nodes palpation, abdominal palpation, respiratory frequency, heartbeats frequency and rectal temperature. Age was estimated based on weight, physical characteristics and reproductive condition. Infants weigh less than 100 g, fur details are not fully defined, and tufts are shorter. Young marmosets weighted between 100 and 250 g, fur details are also not fully defined, and tufts are shorter. Adults weight more than 250 g, fur details are fully defined, and tufts are longer. Pregnant and lactating females were also considered adults. Blood was sampled from the femoral vein and placed in tubes containing clot activators. Volume of sampled blood was under 1% of the marmoset weight. After separation, serum samples were stored at −20 °C until serological evaluation. All animals were tagged with subcutaneous microchips for later identification. After total recovery from sedation, animals were released near the same place of capture inside the parks.

### 2.3 Serological tests

#### 2.3.1 Chikungunya (*a. chikungunya*) IgG and IgM ELISA assays

Anti-*A. chikungunya* IgG or IgM antibodies were detected with two commercial kits (EUROIMMUN, Germany) following manufacturer’s instructions. Sensitized plates were incubated with positive and negative controls, calibrators and serum samples (1:100 dilution; samples were analyzed without replicates due to limited sample availability) for one hour at 37 °C. Secondary antibody (anti-human IgG or IgM conjugated with peroxidase, ready-for-use) was added to each well and incubated for 30 min at room temperature. Chromogenic solution was added to each well and incubated for 15 min at room temperature, followed by the stop solution. Optic density was read at 450 nm. Plates were washed 3 times between each step. All reagents, including positive and negative controls are provided by the kit. The following formula was used to evaluate the results: ratio = sample O.D./calibrator O.D. Samples were considered reactive if ratio was above 1.1.

#### 2.3.2 Zika virus (*O. Zikaense*) IgG and IgM ELISA assays

Detection of anti-*O. zikaense* virus antibodies was by two commercial kits (Vircell, Spain) following manufacturer’s instructions. Sensitized plates were incubated with positive and negative controls, calibrators and serum samples (1:20 dilution; samples were analyzed without replicates due to limited sample availability) for 45 min at 37 °C. Secondary antibody (anti-human IgG or IgM ready-for-use) was added to each well and incubated for 30 min at 37 °C, followed by chromogenic solution for 20 min at room temperature. Stop solution was added and O.D. read at 450 nm. Plates were washed 5 times between incubation steps. All reagents, including positive and negative controls are included in the kit. The following formula was used to evaluate the results: ratio = (sample O.D./mean calibrators O.D.) x 10. Samples with ratio value above 11 were considered reactive.

#### 2.3.3 Dengue virus (*O.Denguei*) IgM ELISA assay

Anti-*O.Denguei* IgM antibodies were detected by a commercial kit (Panbio Dengue IgM Capture ELISA, Abbot laboratories, USA) following manufacturer’s instructions. Positive and negative controls, calibrators and serum samples (1:100 dilution; samples were analyzed without replicates due to limited sample availability) were incubated for 1 h at 37 °C. Then a combination of antigen and antibody detector (anti-human IgM, ready-for-use) was added and incubated for 1 h at 37 °C. Chromogenic solution was added and incubated for 10 min at room temperature and then added the stop solution. Optic density (O.D.) was read at 450 nm. Wells were washed 6 times between each step.

#### 2.3.4 Yellow fever (*O. Flavi*) ELISA assay

96-well plates were incubated with anti-human IgM antibodies, diluted in carbonate buffer at 1:500 dilution, at 4 °C overnight. Then, a PBS solution with 5% Difco and 0.5 Tween20 was added for 30 min at room temperature. Serum samples and controls (positive and negative) at 1:400 dilution were incubated for 60 min at 37 °C. *O. flavi* antigen was added and incubated overnight at 4 °C. Anti-flavivirus conjugate (6B6C-1) at 1:3000 dilution was incubated at 37 °C for 60 min. Then chromogenic TBM solution was added and incubated for 15 min, followed by stop solution. Optic density (O.D.) was read at 450 nm. Samples were considered reactive if O.D. was higher than 0.3.

None of the assays and cutoffs were not formally validated in *C. penicillata*. Samples were not retested. All plate controls met the criteria required by the manufacturers. Sample storage conditions and quality did not affect the results.

### 2.4 RT-PCR

Serum samples were subjected to viral DNA/RNA extraction using an automated protocol on the MagNA Pure 96 System platform (Roche), with the MagNA Pure 96 DNA and Viral RNA kit (Roche), following the manufacturer’s recommendations. After extraction, aliquots of the DNA/RNA were subjected to multiplex RT-qPCR reactions for the detection of viral pathogens, as described below. All RT-qPCR reactions were performed on 7,500 Real-Time PCR Systems thermocyclers (Applied Biosystems), and data analysis was performed using the equipment’s own software.

For *O. zikaense*, *O.Denguei*, and *A. chikungunya* testing, the Kapa Probe Fast qPCR Master Mix kit (Kapa Biosystems) and the primer and probe set provided free of charge by the CDC to LACENS (Trioplex Real-Time RT-PCR Assay kit) were used, as recommended by the manufacturers. This protocol allows for the differential diagnosis of arboviruses through multiplex RT-qPCR reactions for *O. zikaense*, *O.Denguei*, and *A. chikungunya* viruses.

For *O. flavi* testing, the primer and probe set described by Domingo et al. (18) and the Kapa Probe Fast qPCR Master Mix kit (Kapa Biosystems) were used, as recommended by the manufacturer.

For the detection of viruses of the genus Flavivirus, a set of Pan-Flavi primers and probes described by Patel et al. (19) and the Kapa Probe Fast qPCR Master Mix kit (Kapa Biosystems) were used, as recommended by the manufacturer.

### 2.5 Statistical analysis

GraphPad Prism software (version 8.0.1) was used to analyze data. Frequencies were evaluated by Fisher’s exact test. Binomial confidence intervals (95%) of frequencies were calculated using an online calculator (https://statpages.info/confint.html).

## 3 Results

To evaluate arbovirus circulation, 72 serum samples from 68 free-ranging black-tufted marmosets were submitted to serological evaluation. Two animals (2/68, 2.94% [95% binomial CI = 0.36 to 10.22%]) were seroreactive by the *A. chikungunya* anti-IgM assay. One of them was evaluated in two different periods of capture, with three months of interval between samplings. Both samples from the recaptured *A. chikungunya*-reactive animal were also seroreactive by the *O. zikaense* anti-IgM assay. Frequency of putative seroreactivity for *O. zikaense* (IgM) was 1.47% (1/68 [95% binomial CI = 0.04 to 7.92%]). The *A. chikungunya* and *O. zikaense*-reactive animal was an adult male, and the *A. chikungunya*-reactive was an adult female. Both animals were captured in *Parque das Mangabeiras*, both samplings of the male were in 2022 (April and July), and the female was captured in 2023 (September).

All serum samples were non-reactive for *A. chikungunya* anti-IgG, *O.Denguei* anti-IgM, *O. zikaense* anti-IgG and *O. flavi* anti-IgM. The low frequency of seroreactivity prevented an appropriate comparisons between sexes and age categories so difference between sex or estimated age was found. Table 1 details information about the seroreactive animals.

**Table 1:** Free-ranging *Callithrix penicillata* seroreactive samples and animals to arboviruses.

ID	Park	PC*	Age	Sex	CHIKV-IgM (OD/ratio)	CHIKV-IgG (OD/ratio)	DNV-IgM (OD/ratio)	ZKV-IgM (OD/ratio)	ZKV-IgG (OD/ratio)	YFV-IgM (OD/ratio)
C001	PM	April 2022	A	M	R (1.515/4.647)	NR (0.014/0.036)	NR (0.178/0.293)	R (2.161/21.682)	NR (0.173/1.922)	NR (0.073)
C001	PM	July 2022	A	M	R (1.514/4.644)	NR (0.002/0.005)	NR (0.128/0.211)	R (1.26/12.641)	NR (0.023/0.255)	NR (0.067)
C072	PM	Sept. 2023	A	F	R (0.399/1.223)	NR (0.011/0.028)	NR (0.23/0.379)	NR (0.477/4.785)	NR (0.029/0.322)	NR (0.081)

ID, animal identification; PC, period of capture; A, adult; M, male; F, Female; CHIKV-IgM, A. chikungunya anti-IgM assay (reactive if ratio above 1.1); CHIKV-IgG, A. chikungunya anti-IgG assay (reactive if ratio above 1.1); DNV-IgM, O.Denguei anti-IgM assay (reactive if ratio above 1.1); ZKV-IgM, O. zikaense anti-IgM assay (reactive if ratio above 11); ZKV-IgG, O. zikaense anti-IgG assay (reactive if ratio above 11); YFV-IgM, O. flavi fever anti-IgM assay (reactive if O.D. above 0.3); PM, Parque das Mangabeiras; A, adult; R, reactive; NR, not reactive.

*None of these samples were available for RT-PCR.

From the 68 captures black-tufted marmosets, 16 provided serum samples that were also sufficient for performing RT-PCR to detect *O. zikaense, O.Denguei*, *O. flavi*, *A. chikungunya* RNA and to a pan-flavivirus assay. All 16 samples were negative, but not all animals were tested due to limited sample availability and seroreactive animals were not tested by RT-PCR. Serum samples from the two seroreactive marmosets were not available for RT-PCR.

## 4 Discussion

This study investigated serological evidence of circulation of arboviruses in free-ranging black-tufted marmosets (*C. penicillata*) from urban or periurban areas. Serological response was low with only two animals (2.94%) seroreactive to *A. chikungunya* and one (1.47%) reactive to *O. zikaense*. Both seroreactive animals were from *Parque das Mangabeiras* (PM), the largest park in the city of Belo Horizonte with 236 hectares, and delimited by residential areas and by a mining area. Frequency of putative seroreactivity to arboviruses was low (2.94%) as reported by other studies with neotropical primates (14–16).

Neutralizing antibodies against *A. chikungunya* and *O. zikaense* were found in 2.9% of callithrichids from northeastern region of Brazil, one common marmoset (*C. jacchus*) was seroreactive to *A. chikungunya* and one black-tufted marmoset reactive to *O. zikaense* (16). Favoretto et al. (13) investigated *O. zikaense* infection in peridomestic common marmosets in northeastern Brazil (State of Ceará) and one animal (1.8%, 1/55) had neutralizing antibodies against *O. zikaense*.

More recently Dias et al. (20) reported serological response, evaluated by hemagglutination inhibition, to several arboviruses in free-ranging common marmosets in northeastern Brazil (State of Sergipe). Animals were seroreactive to Mucambo virus (*Alphavirus mucambo*) (2/50), *O. flavi* (21/50), Ilheus virus (*Orthoflavivirus ilheusense*) (18/50), *O. louisense* (24/50), Rocio virus (21/50), *O. nilense* (20/50), Cacipacore virus (*Orthoflavivirus cacipacoreense*) (5/50), *O. zikaense* (1/50) and *O.Denguei* four serotypes (11/50). Bandeira et al. (21) reported one free-ranging common marmoset from northeastern Brazil (State of Pernambuco) with antibodies to *O. louisiense*, *O. nilense* and *O.Denguei* serotype 4.

Contrasting with those results, La Serra et al. (22) had no serological reactive free-ranging neotropical primates to *A. chikungunya* or Mayaro virus (*Alphavirus mayaro*). However, through molecular tests they identified black-tufted marmosets positive for *O.Denguei* serotypes 1 (1/209) and 3 (2/209), *O. flavi* (1/209), *O. louisense* (7/209) and *O. zikaense* (8/209). Here, all samples submitted to molecular assays were negative. Importantly, the Plaque Reduction Neutralization Test (PRNT) is a more accurate indicator of specific immune responses. Consequently, its results are not directly comparable with those obtained from broad serological screening assays or molecular detection methods, which measure different biological parameters.

In 2022, Minas Gerais had 70,421 confirmed human cases of dengue infection, 6,382 of *A. chikungunya* infection and 18 of *O. zikaense* infection (23). *A. chikungunya* cases occurred mostly during two periods of the year, between March and April, and in December. *O. zikaense* infection in humans was less frequent than other arboviruses in this year and was distributed throughout the year. One animal was captured and evaluated two times in 2022, both times it was seroreactive to *A. chikungunya* and *O. zikaense* with IgM response in this animal persisting at least until July for both viruses. The first capture was in April, at the same time *A. chikungunya* was circulating in the human population. The other animal was captured in September of 2023 and was seroreactive to *A. chikungunya*. In this year, human *A. chikungunya* cases were concentrated between February and May (24), which were before diagnosis in the marmoset. It is noteworthy that, although persistent IgM reactivity over several months is a plausible interpretation, this finding also raises concerns regarding assay specificity, nonspecific binding, and cross-reactivity, all of which should be considered as potential confounding factors. Although we found no significant differences in positivity frequency between sexes or among age groups, the low number of positive cases limited the statistical power of these comparisons. Therefore, these findings should be interpreted with caution.

Arboviruses can cross-react with each other in serological tests, which is more commonly observed between *O. zikaense* and *O.Denguei* viruses. However, other flaviviruses can cross-react with each other and with *A. chikungunya* (25–27). Serologic studies with neotropical primates demonstrated cross-reactivity between *O.Denguei* and other flaviviruses (*O. zikaense*, *O. nilense* and *O. louisense*) (15, 16). Based on known cross-reactivity between these viruses, serological assays have limited specificity to determine which virus induced that serological response. One of the marmosets evaluated in this study had IgM against both *A. chikungunya* and *O. zikaense*, but it is impossible, based on serology, to determine which of these viruses triggered this antibody response, and exposure to both viruses cannot be ruled out as well. This limitation could have been addressed by performing a PRNT. However, this was not feasible in the present study because the limited volume of available serum samples precluded additional testing.

Yellow fever is an important cause of death to neotropical primates (8, 9). Frequency of death in marmosets associated with *O. flavi* infection is lower than other species. In this study all marmosets evaluated were non-reactive in the *O. flavi* serological assay. Assays to determine the presence of neutralizing antibodies would be useful to access their susceptibility in a new outbreak.

ELISA assays employed in this study were based on the detection of specific antibodies using anti-human Ig secondary antibodies. Importantly, previous studies conducted by our group and others have demonstrated that anti-human Ig cross-reacts with specific antibodies from various neotropical primate species (28–30). Moreover, these assays yielded results comparable to those obtained with serological methods that do not require secondary antibodies (28, 29), supporting the reliability of this approach for antibody detection in these species.

In conclusion, frequency of putative seroreactivity to arboviruses in free-ranging black-tufted marmosets was low in an area where arboviruses are endemic in the human population. Furthermore, arboviruses genomic sequences were not detected in serum samples, but seroreactive animals were not tested by RT-PCR due to sample limitation.

## Data Availability

The original contributions presented in the study are included in the article/supplementary material, further inquiries can be directed to the corresponding author/s.
